# Centralized Communication of Blood Culture Results Leveraging Antimicrobial Stewardship and Rapid Diagnostics

**DOI:** 10.1093/ofid/ofz321

**Published:** 2019-07-15

**Authors:** Shelby Shemanski, Nicholas Bennett, Cynthia Essmyer, Kevin Kennedy, Donna M Buchanan, Andrew Warnes, Sarah Boyd

**Affiliations:** 1 Saint Luke’s Pharmacy, Saint Luke’s Health System, Kansas City, Missouri; 2 Antimicrobial Stewardship Program, Saint Luke’s Health System, Kansas City, Missouri; 3 Department of Microbiology, Saint Luke’s Health System, Kansas City, Missouri; 4 Department of Research, Saint Luke’s Health System, Kansas City, Missouri; 5 Research Administration, Department of Biomedical and Health Informatics, University of Missouri-Kansas City School of Medicine, Saint Luke’s Health System, Kansas City, Missouri; 6 Infectious Diseases, Saint Luke’s Health System, Kansas City, Missouri

**Keywords:** antimicrobial stewardship, rapid diagnostics

## Abstract

**Objective:**

This study aimed to determine if integrating antimicrobial stewardship program (ASP) personnel with rapid diagnostic testing resulted in improved outcomes for patients with positive blood cultures.

**Method:**

Beginning in 2016, Saint Luke’s Health System (SLHS) implemented a new process where all positive blood cultures were communicated to ASP personnel or SLHS pharmacy staff. Pharmacists then became responsible for interpreting results, assessing patient specific information, and subsequently relaying culture and treatment information to providers. This was a multisite, pre-post, quasi-experimental study (Pre: August to December 2014; Post: August to December 2016). Patients 18 years of age and older with a positive blood culture during admission were included (2014, n = 218; 2016, n = 286). Coprimary outcomes of time to optimal and appropriate therapy were determined from time of culture positivity via gram stain. Secondary outcomes focused on clinical, process, and fiscal endpoints. A pre-post intervention physician survey was conducted to assess the impact on antimicrobial decision making and perceived effect on patient outcomes.

**Results:**

There was no difference in time to appropriate therapy groups (*P* = .079). Time to optimal therapy was 9.2 hours shorter in 2016 (*P* = .004). Provider surveys indicated the process improved communication among clinicians and facilitated a shared decision-making process with a perceived improvement in patient care.

**Conclusions:**

An ASP-led blood culture communication process for patients with positive blood cultures was shown to improve time to optimal therapy, support physicians in their decision making on critical lab data, and improve the care for hospitalized patients.

## Teaser

Integration of antimicrobial stewardship personnel plus rapid identification testing was associated with reduced time to optimal therapy, increased collaboration between clinical staff, and improved care for hospitalized patients with positive blood cultures.

## INTRODUCTION 

Bloodstream infections (BSI) represent a significant burden to health care systems and are associated with increased morbidity and mortality [1]. The emergence of newer rapid diagnostic testing (RDT) platforms, such as polymerase chain reaction (PCR) and matrix-assisted laser desorption ionization time-of-flight (MALDI-TOF), have allowed for earlier streamlining of therapies for various infection sources. With the continued evolvement of RDTs, it is essential to have clinicians with a detailed understanding of testing platforms be actively involved in the dissemination, interpretation, and therapeutic approaches of such results. 

A number of studies have demonstrated the benefits to integrating antimicrobial stewardship program (ASP) and rapid identification (ID) technologies; however, they either have been single center or, if multisite, they leverage site-specific practitioners or evaluate specific pathogens (eg, gram negative, candidemia). One example of a multisite study by Box et al found that ASP and non-ASP pharmacist input at 5 community hospitals for patients with gram-positive bacteremia reduced time to optimal therapy [2]. Of the few randomized studies, Banerjee et al prospectively evaluated PCR or MALDI-TOF plus ASP input compared to traditional testing and PCR testing with templated micro comments. ASP input plus PCR testing had the greatest effect on time to de-escalation of therapy [3]. Additional studies, mostly quasi-experimental, found reduced time to pathogen ID, hospital length of stay, mortality, and costs relative to combined ASP with rapid ID interventions [4–13]. A recent cost effectiveness model suggests that RDTs for BSIs had nearly doubled the likelihood of being cost effective when coupled with ASP services, thus reducing healthcare costs [14]. An additional meta-analysis has solidified the value of ASP services in interpreting and disseminating RDT results [15].

Limited data exist for the combination of RDTs along with a centralized blood culture communication process for a multihospital setting using both ASP members and other non-ASP pharmacists. The aim of this study was to assess the impact of combining RDT testing for BSIs supported by ASP and non-ASP pharmacist input.

## METHODS

### Study Design

This multicenter, quasi-experimental study was conducted at Saint Luke’s Health System and received investigational review board waiver approval. Saint Luke’s is a 10-hospital health system representing academic, community, and critical access hospitals. Five of the 10 hospitals were included in this study: (1) Saint Luke’s Hospital [academic], 431 beds; (2) Saint Luke’s East [community], 201 beds; (3) Saint Luke’s North [community], 159 beds; (4) Saint Luke’s South [community], 125 beds; (5) Saint Luke’s Cushing [community], 54 beds. ASP personnel are available to each site between 7:00AM and 4:00PM Monday through Friday, and infectious disease providers also are available at each site for consult services 7 days a week. Our integrated practice model involves decentralized pharmacists, who provide direct patient care 5 days a week during daytime hours at all but 1 hospital, while centralized pharmacists provide patient care at all other times of the day. Patients with any treated BSI identified using PCR, MALDI-TOF, or conventional testing during a 5-month period (August 1, 2016 to December 31, 2016, Figure 1) were compared to a historical cohort with a treated BSI identified using PCR or conventional testing 2 years prior (August 1, 2014 to December 31, 2014). Patients were included if 18 years and older with a positive blood culture for which treatment was pursued for the isolated organism. Only the initial positive culture was included in the organism analysis, though all organisms were considered for determination of appropriate or optimal therapies. All positive isolates (eg, fungal, bacterial) were allowed. Exclusion criteria included patients not admitted (emergency room only or observation), blood cultures deemed contaminants (did not receive any treatment), receiving less than 48 hours of antimicrobial therapy, discharge prior to culture turning positive, or transfer from a non-Saint Luke’s hospital with BSI. 

**Figure 1.**
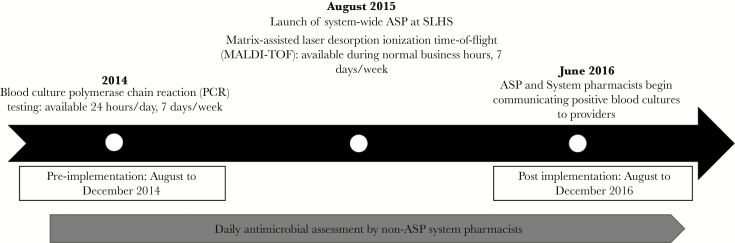
Project Implementation Timeline

### Baseline Characteristics

Baseline patient characteristics were obtained from the electronic medical record (EMR) and by extracting International Classification of Diseases (ICD)-9 (2014) [16] and ICD-10 (2016) [17] codes. 

### Microbiology Workflow

All obtained blood cultures are sent to a central microbiology lab located at the academic medical center. In 2014, the lab implemented the BioFire Diagnostics’ FilmArray Blood Culture Identification Panel (Salt Lake City, Utah) for initial identification (ID) of isolates from positive blood cultures. This testing platform is a multiplex PCR that can detect 19 bacteria species, 5 yeast species, and 3 resistance genes. Gram stain is performed on all cultures and triaged to Cepheid Xpert MRSA/SA blood culture (Sunnyvale , California) PCR if there is suspicion of *Staphylococcus* species (aerobic bottle only). All other isolates are tested via FilmArray. In 2015, the lab added Bruker’s MALDI-TOF (Billerica, Massachusetts) as an alternative method for organism ID from solid culture media. All positive blood cultures are first run on PCR. If no specific ID is determined by PCR, further ID is performed by conventional workup (2014) or by MALDI-TOF (2016). Blood culture samples are run on PCR 24 hours a day, 7 days a week, and MALDI-TOF is run 7 days a week during normal business hours (approximately 7:00AM to 3:30PM). Susceptibility testing was performed, but it was only included in the study results if it was performed at Saint Luke’s Hospital and if it did not require send out to another reference laboratory. 

### Pre-intervention Period—No ASP or Pharmacy Involvement

During the pre-intervention period, microbiology staff contacted nursing with positive blood cultures (gram stain or PCR), who subsequently communicated results to the treating physician. Passive pharmacist response occurred during this period as there was no mechanism to alert them to positive culture results, except for select EMR-based drug-bug mismatch alerts, which were only available once susceptibilities were reported. As part of their daily clinical responsibility, all patients had a non-ASP pharmacist evaluating appropriateness of antimicrobial therapy in both groups.

### Intervention Period—Direct Communication by ASP and Pharmacy Staff

Saint Luke’s Health System ASP is a centralized program launched in August 2015 that covers all system hospitals utilizing the integrated EMR or direct communication. Staffing at the time included 1 infectious diseases full time ASP pharmacist and a 0.5 infectious diseases physician who spends half her time doing ASP work. Beginning in June 2016, all new positive blood culture results (gram stain or PCR) or MALDI-TOF results were communicated by microbiology staff to the ASP pharmacist Monday through Friday, 7:00AM to 3:30PM, and to non-ASP pharmacists and pharmacy residents at the academic hospital all other hours. Non-ASP staff were provided a 1-hour webinar training and guidance document regarding treatment options for select pathogens. Calls were centralized due to staffing and workload limitation at nonacademic sites. By doing so, it kept the number of pharmacists involved in the process lower, thus minimizing practice variance. Site-based pharmacists were involved when needed to facilitate appropriate changes. After detailed patient review, pharmacists would contact the appropriate treating provider with culture results and suggest therapy changes, if appropriate. Pharmacists then placed a “critical notification” progress note in the medical record that included the name of the physician contacted, date and time of communication, and blood culture results. 

Pharmacists recorded blood culture communication information into a database, including the time of communication, provider contacted, relevant culture updates, and other supporting information. All cultures and communications were reviewed by ASP staff the following day or on the Monday following a weekend.

### Outcomes

The coprimary outcomes of this study were time to appropriate therapy and time to optimal therapy. Time to appropriate therapy was defined as the administration of the first antimicrobial with known susceptibility to the isolated organism. Time of culture positivity (via gram stain) served as time zero for relevant endpoints, as this represents the point in time during which clinicians began to have useful objective information to make antimicrobial decisions. Thus, values for time to appropriate therapy are represented as negative numbers. Time to optimal therapy was time to most appropriately narrow therapy based on isolated organism(s), susceptibility data, and need for coverage of concurrent suspected or confirmed infections. All therapies were reviewed by 2 pharmacists. If discrepancies existed, cases were reviewed by an infectious disease physician. Secondary outcomes included time to organism ID, time to organism susceptibility, length of stay, intensive care unit length of stay (if applicable), inpatient mortality, inpatient and antimicrobial costs, rates of recommendation acceptance, and provider satisfaction regarding the process change.

Physician satisfaction with blood culture communication at both pre- and postintervention was assessed. Provider groups included in the survey were hospitalists or internal medicine, intensivists, medical residents, surgeons, and infectious disease. Respondents were asked to characterize the number of blood culture calls received per month, timeliness of communication, adequacy of case-specific information, beliefs about the communication process improving outcomes and affecting resistance, and shared decision making.

### Statistical Analysis

Data are shown as median + interquartile range for continuous and number (%) for categorical and are tested with *t* and χ ^2^ tests where appropriate. Because many of our outcomes are nonnormal and skewed right, we chose to model these outcomes using median regression as it is less impacted by nonnormal data. In these models, we estimated the independent association between our primary exposure variable (pre- and postintervention) and our outcomes of interest by adjusting for the following: age, sex, race, diabetes, acute kidney injury, chronic kidney disease, stroke, congestive heart failure, chronic lung disease, chronic obstructive pulmonary disease, an immunosuppressed condition, cancer, myocardial infarction, need for renal replacement therapy, infection source, intensive care unit (ICU) admission, and academic versus community site. In the pre-intervention cohort, ICD-9 codes were used to define the above adjusted variables and ICD-10 codes were used in the postintervention cohort. All analysis was done with SAS version 9.4 (Cary, NC). 

## RESULTS

**Figure 2.**
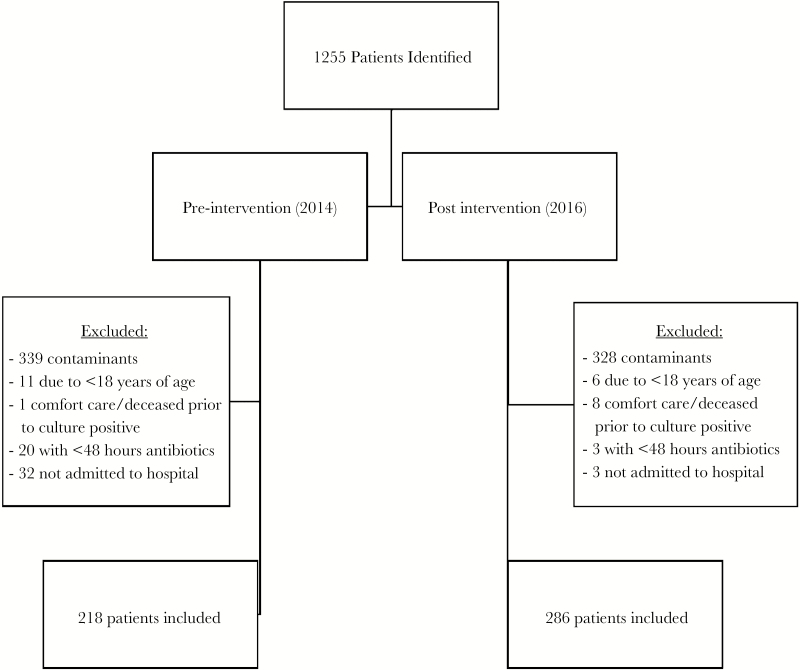
Flow of Study Inclusion and Exclusion

A total of 1255 patients were identified during the study period, of which 504 met inclusion criteria (n = 218 in 2014; n = 286 in 2016; Figure 2). Baseline characteristics were similar between both groups (Table 1). Nearly 50% of patients in both groups were treated at our academic medical center. There were fewer ICU admissions in the 2016 group (44.5% vs 35.3%; *P* = .036). There were more line-associated, foreign device, and endocarditis BSIs in 2014, which paralleled the higher rates of *Staphylococcus aureus* found in that group. In 2016, there were higher rates of genitourinary infections and *E. coli* infections. Sources of BSIs and incidence rates of specific organisms also are depicted in Table 1 (and Supplementary Table 1). 

**Table 1.** Baseline Characteristics

Characteristics	Pre-intervention (2014; n = 218)	Post-intervention (2016; n = 286)	*P* value
Age	65.4 ± 17	65.4 ± 17	.680
Male, no. (%)	114 (52.3)	150 (52.4)	.900
Patient race, no. (%)			.155
Caucasian	167 (76.6)	226 (79.1)	
Non-Caucasian	51 (23.4)	60 (20.9)	
Location, no. (%)			.406
Saint Luke’s Hospital	108 (49.8)	127 (44.6)	
Saint Luke’s Cushing	6 (2.7)	8 (2.8)	
Saint Luke’s East	50 (22.8)	87 (30.3)	
Saint Luke’s North	34 (15.4)	44 (15.3)	
Saint Luke’s South	20 (9.1)	20 (7)	
Comorbidities, no. (%)			
Diabetes mellitus	81 (37.3)	110 (38.6)	.771
Acute kidney injury	65 (30.0)	93 (32.6)	.522
Chronic kidney disease	64 (29.5)	73 (25.6)	.333
Heart failure	47 (21.7)	45 (15.8)	.092
Chronic obstructive pulmonary disease	37 (14.1)	35 (12.3)	.862
Myocardial infarction	26 (12)	40 (14)	.499
ICU admission, no. (%)	97 (44.5)	101 (35.3)	.036
Need for RRT (%)	18 (8.3)	17 (6)	.309
Bloodstream infection source, no. (%)			.037
Genitourinary	53 (24.3)	93 (32.5)	
Line associated/foreign device/endocarditis	46 (21.1)	37 (12.9)	
Intra-abdominal	33 (15.1)	41 (14.3)	
Other/unknown	31 (14.2)	28 (9.8)	
Pulmonary	20 (9.2)	26 (9.1)	
Skin and soft tissue/bone-joint	35 (16.1)	61 (21.3)	

Abbreviations: ICU, intensive care unit; RRT, renal replacement therapy.

**Figure 3.**
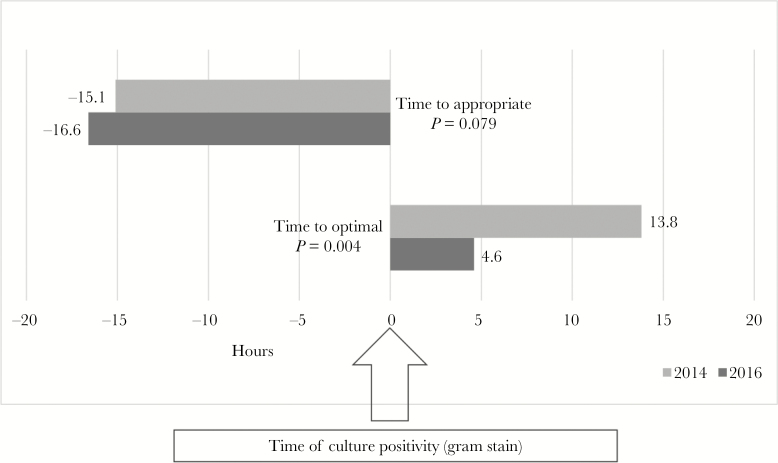
Median Time to Appropriate and Optimal Antimicrobials

All cases were reviewed separately by 2 pharmacists, and only 3 discrepancies existed requiring review from an infectious disease physician. The coprimary outcome of time to appropriate antimicrobial therapy was not significantly different between the 2014 and 2016 groups (-16.6 vs -15.1 hours from gram stain positivity; *P* = .079, Figure 3). Time to optimal therapy was significantly shorter by 9.2 hours in 2016 (13.8 vs 4.6 hours; *P* = .004, Figure 3). When stratified based on hospital type (academic vs community), there appeared to be a slightly more pronounced time to optimal therapy at the academic center (SupplementaryAppendix’s Tables 3–4). 

Shorter time to organism ID from PCR testing was noted in 2014 (1.4 vs 1.5 hours; *P* = .01, Table 2). The majority of positive cultures were identified via PCR; 97% in 2014 and 87% in 2016. The median time to organism ID with MALDI-TOF (2016 only) was 25.5 hours. Susceptibility information was reported sooner in the 2014 group (43.8 vs 48.7 hours; *P* < .001). There was no difference in inpatient mortality (4.1% vs 4.5%; *P* = .82) or total length of hospital stay (*P* = .23). 

**Table 2.** Primary and Secondary Outcomes

Outcome	2014 (n = 218)	2016 (n = 286)	*P* value
Time to appropriate therapy, hours from gram stain, median (IQR)	-15.1 (-21.3 to -1.6)	-16.6 (-24.6 to -6.6)	.079
Time to optimal therapy, hours from gram stain, median (IQR)	13.8 (-12.3 to 48)	4.6 (-16.7 to 35.6)	.004
Time to organism identification from gram stain–PCR, hours, median (IQR)	1.4 (0.1–1.8)	1.5 (0.1–2.1)	.010
Time to organism identification from gram stain–MALDI-TOF, hours, median (IQR)	—	25.5 (17.7–33.9)	—
Time to susceptibility, hours, median (IQR)	43.8 (34.6–50.1)	48.7 (39–57.4)	<.001
ICU length of stay, days, median (IQR)	3.0 (2.0, 8.0)	3.0 (2.0, 6.0)	.081
Hospital length of stay, days, median (IQR)	7 (5–13)	7 (5–11)	.228
Inpatient mortality, no. (%)	9 (4.1)	13 (4.5)	.820
Hospital length of stay from positive gram stain, days, median (IQR)	6 (4–10)	6 (4–10)	.104
Inpatient cost, $, median (IQR)	15 475 (7779–29 636)	14 884 (8984–27 667)	.803
Antimicrobial costs, $, mean	426.32	265.96	N/A

Abbreviations: ICU, intensive care unit; IQR, interquartile range; MALDI-TOF, matrix assisted laser desorption ionization-time of flight; N/A, not applicable; PCR, polymerase chain reaction.

Hospitalization costs were lower in the 2016 group, although no statistically significant difference existed ($14 884 vs $15 475; *P* = .792, Table 2). Antimicrobial costs were obtained from our drug wholesaler and reflect acquisition costs based on 2017 cost data, which was applied to both groups to ensure accurate comparison. Direct antimicrobial costs were $160 less per patient in 2016, representing an estimated $110 000 in annualized savings from drug optimization. 

In our fully adjusted multivariable model, our results showed a significant 6.65 hour reduction in time to optimal therapy in 2016 (*P* = .0393). No difference was found in time to appropriate therapy. Organism ID via gram stain and time to susceptibilities were significantly longer in the 2016 cohort by 2.61 (*P* = .0008) and 7.3 hours (*P* < .0001), respectively (Supplementary Appendix’s Table 2). 

Of the 252 recorded communications between pharmacists and physicians in the postintervention group, 132 resulted in no changes to therapy, 81 recommended escalation of therapy or dose optimization, and 39 were for de-escalation. All recommendations for no change and escalation or dose modification were accepted by physicians. Of the 39 proposed de-escalation attempts, 31 (79.5%) were accepted. Of the 8 de-escalation attempts not accepted, 7 of 8 occurred during after-hour periods (outside 7:00AM to 4:00PM) and 3 of 8 occurred at the academic hospital. The 112 documented after-hour calls primarily were communicated to hospitalists or internal medicine (79.6%) and ICU providers (16.1%). 

A physician survey was completed pre-implementation in 2016 (n = 47) and 6 months after the change in 2017 (n = 41). Several differences in responses were noted between the 2 surveys (Table 3). When asked if they were being provided with an adequate amount of case-specific information needed to determine appropriate treatment, there were significantly more providers who agreed or strongly agreed with this statement after the process change (Strongly agreed, 21.6% postintervention vs 2.3% pre-intervention; *P* = .020). Trending, nonsignificant findings included perceived improvement in patient outcomes and resistance (*P* = .088), and a shift towards a shared decisions (pharmacist plus provider) regarding treatment of culture results (*P* = .057). 

**Table 3.** Provider Survey

Question	Response	Pre-intervention (n = 47)	Postintervention (n = 41)	*P* value
On average, how often are you contacted in a month about a positive blood culture for a hospitalized patient?	0–1	29 (61.7%)	24 (58.5%)	.916
	2–4	13 (27.7%)	13 (31.7%)	
	≥5	5 (10.6%)	4 (9.8%)	
Which category best describes you?	Resident	16 (34.8%)	7 (17.1%)	.289
	Hospitalist/internal	18 (39.1%)	17 (41.5%)	
	Medicine	6 (13.0%)	10 (24.4%)	
	Intensivist	3 (6.5%)	2 (4.9%)	
	Surgeon	3 (6.5%)	5 (12.2%)	
	Infectious disease	No response = 1		
Positive blood culture results of hospitalized patients are communicated in a timely manner.	Strongly disagree	1 (2.3%)	2 (5.3%)	.263
	Disagree	1 (2.3%)	1 (2.6%)	
	Somewhat disagree	4 (9.3%)	1 (2.6%)	
	Neutral	4 (9.3%)	4 (10.5%)	
	Somewhat agree	7 (16.3%)	3 (7.9%)	
	Agree	22 (51.2%)	16 (42.1%)	
	Strongly agree	4 (9.3%)	11 (28.9%)	
		No response = 4	No response = 3	
When I am notified about a hospitalized patient’s positive blood culture result, I am provided with an adequate amount of case-specific clinical information needed to determine appropriate antimicrobial treatment.	Strongly disagree	2 (4.7%)	1 (2.7%)	.020
	Disagree	2 (4.7%)	4 (10.8%)	
	Somewhat disagree	7 (16.3%)	3 (8.1%)	
	Neutral	11 (25.6%)	3 (8.1%)	
	Somewhat agree	4 (9.3%)	8 (21.6%)	
	Agree	6 (37.2%)	10 (27.0%)	
	Strongly agree	1 (2.3%)	8 (21.6%)	
		No response = 4	No response = 4	
The current communication process about positive blood culture results of hospitalized patients supports improved patient outcomes and helps minimize antimicrobial resistance.	Strongly disagree	0 (0.0%)	3 (7.9%)	.088
	Disagree	3 (7.0%)	0 (0.0%)	
	Somewhat disagree	5 (11.6%)	1 (2.6%)	
	Neutral	13 (30.2%)	11 (28.9%)	
	Somewhat agree	6 (14.0%)	4 (10.5%)	
	Agree	14 (32.6%)	13 (34.2%)	
	Strongly agree	2 (4.7%)	6 (15.8%)	
		No response = 4	No response = 3	
With the current communication process about positive blood culture results of hospitalized patients, who makes decisions about antimicrobial treatment?	Myself alone	3 (7.1%)	0 (0.0%)	.57
	Mostly myself	22 (52.4%)	12 (31.6%)	
	Myself with a pharmacist or infectious disease physician (equally shared decision)	14 (33.3%)	23 (60.5%)	
	Mostly a pharmacist or infectious disease physician	3 (7.1%)	2 (5.3%)	
	A pharmacist or infectious disease physician alone	0 (0.0%)	1 (2.6%)	
		No response = 5	No response = 3	
In the case of positive blood culture results of hospitalized patients, who do you prefer make decisions about antimicrobial treatment?	Myself alone	2 (4.8%)	2 (5.3%)	.879
	Mostly myself	10 (23.8%)	9 (23.7%)	
	Myself with a pharmacist or infectious disease physician (equally shared decision)	26 (61.9%)	22 (57.9%)	
	Mostly a pharmacist or infectious disease physician	4 (8.5%)	4 (10.5%)	
	A pharmacist or infectious disease physician alone	0 (0.0%)	1 (2.6%)	
		No response = 5	No response = 3	

## DISCUSSION

Previous studies found decreased time to effective therapy, length of stay (LOS), and mortality with implementation of RDT for BSI, but only when ASP was assisting [4–12]. Our study supports this finding with decreased time to optimal therapy, while also showing improved provider satisfaction with the enhanced blood culture communication change. Our study also shows the value of a centralized ASP-led, pharmacist-driven blood culture communication for all positive blood culture results, not just those detected using RDTs. This represents a unique shift from previous studies that primarily focused only on RDT-positive results.

We found that communication by pharmacists led to patients being on optimal therapy 9.2 hours sooner, using time zero as the time at which gram stain results were available. Outcomes appear to be similar when stratified by our academic center versus community hospitals, though the difference pre- and postimplementation was slightly more pronounced at the academic center. Many patients were on appropriate broad-spectrum coverage for isolated organisms prior to time of gram stain positivity, suggesting appropriate initial therapy selection in both groups. Given that PCR testing was available in both groups, pharmacist input is the primary driver for the change. The adjusted analysis showed that pharmacist involvement still was associated with a significant reduction in time to optimal therapy (6.65 hours). 

Despite the availability of MALDI-TOF in addition to PCR testing in the postintervention group, the median time to ID using MALDI-TOF was 25.5 hours, which is well beyond the average time to therapy optimization (4.6 hours in the post intervention group). Additionally, the majority of organisms were identified by PCR testing in this group, further supporting the argument that the pharmacist-driven process led to the outcomes of interest. Time to susceptibility was significantly shorter in the pre-intervention group; however, this did not affect time to optimal or appropriate therapy. In fact, the longer time to susceptibility information in the postintervention group further supports the essential role of the pharmacist to influence patient care decisions. Our results fall in line with other literature suggesting that the most effective use of RDT is by putting the results in the hands of clinicians with a working understanding of RDT output and limitations [18]. Our results did not show an effect on mortality or LOS. 

Documented discussions between physicians and pharmacists found a high rate of agreement for antimicrobial therapies based on culture results, especially when escalation of therapy was necessary. These findings correlate with the physician survey findings. A greater degree of hesitancy existed when de-escalation suggestions were made, with 31 of 39 (79.5%) being accepted. This could partially be due to calls made outside of normal daytime hours, when the physician notified was not the primary provider caring for the patient and wished to defer therapy decisions to the day team. Overall, 120 of 252 (46.7%) of recorded initial culture communication discussions resulted in some type of therapy change with a high acceptance rate (93.3%).

Our study provides the second known adjunctive provider survey regarding a practice change to an ASP-led BSI communication, intended to assess physician views on how ASP and RDT-enhanced blood culture communication changes impact care [19]. Provider survey trends support physicians receiving greater patient-specific details (especially if the physician is on-call), preferring shared decision making when reacting to BSI cultures, and a belief that the process improves outcomes and contributes to reducing resistance development. Any process change that supports physicians in making critical decisions is a positive step forward in patient care.

We believe our study had several design strengths. First, we centralized communication for multiple hospitals to a focused group of pharmacists. Resource-limited hospitals or health systems can incorporate non-ASP pharmacy clinicians to provide around-the-clock blood culture triaging. Centralizing our communication minimized the practice variance with recommendations particularly with guidance documents and education on handling specific culture or RDT results. We estimate that each call, depending on the complexity, required 10 to 15 minutes of pharmacist time to review relevant patient case information and contact the necessary provider. We have been able to sustain this service with our current 24 hours a day, 7 days a week coverage plan and average roughly 4 to 5 blood culture calls per day. Second, because PCR testing was established in the pre-intervention group, the major driver of the decreased time to optimal therapy was due to pharmacist input. Further solidifying this point is that there was a reduction in antimicrobial costs suggesting a greater emphasis on de-escalation with active pharmacist-physician discussions. We excluded contaminated blood cultures, which may have provided additional therapy optimization and cost savings potential with therapy avoidance. Third, as a standard of care both prior to and while implementing centralized blood culture communication, our system pharmacists evaluate appropriateness of antimicrobial therapy as part of required daily patient monitoring, supporting the idea that the enhanced communication method further advanced the ability to make therapy modifications. This also may partly explain why the differences in time to optimal therapy may have been shorter as compared to other studies. 

The study did have limitations. First, the retrospective quasi-experimental design did not allow for randomization and is subject to biases related to confounding variables. Notably there were differences in types of infections in each group with an increase in genitourinary and skin and soft tissue or bone-joint infections and a decrease in line, device, or endocarditis sources in the 2016 group. However, a reasonable argument could be made that line or device-related infections are more predictable in their infectious cause, potentially leading to early appropriate therapy initiation and streamlining. Second, there is subjectivity related to time to optimal and appropriate treatment for the primary outcomes; however, we attempted to account for this by having multiple personnel review the data. Third, although the study represents multiple hospitals, they are all in the same health system and geographic region, thus, may not reflect practice and resistance patterns to standard therapies in other regions. Fourth, data collection began within 2 months after implementation of the communication change that may have affected endpoints, such as likelihood of therapy narrowing due to provider unfamiliarity with the change. Lastly, the shorter pre- and postintervention periods used in this study may limit the inclusion of a wider array of isolates encountered throughout a year.

To our knowledge this is the first study to support the implementation of a centralized blood culture communication for all culture results, not just RDTs, for a multihospital health system. It adds to the literature supporting integration of RDT into ASP workflows by improving time to optimal therapy for patients with BSI. The results are unique for 3 reasons: (1) the use of combined ASP and non-ASP pharmacists and residents enhanced the ability to receive, assess, and discuss positive blood culture results with physicians; (2) results further support the idea that RDTs are most useful when coupled with clinicians who have an in-depth understanding of testing output and utility; and (3) our unique provider surveys suggest enhancement of care from a provider’s perspective when utilizing a pharmacist-driven blood culture communication model. 

In conclusion, a centralized ASP-led, non-ASP pharmacist-supported blood culture communication process for patients with BSI was shown to improve time to optimal therapy, support physicians in their decision making on critical lab data, and improve the care provided in hospital settings. These results can be used to support integration of RDTs with ASP and non-ASP pharmacist input to help providers make more timely therapeutic choices and to deploy such services, even in resource-limited settings. 

## Supplementary Data

Supplementary materials are available at *Open Forum Infectious Diseases* online. Consisting of data provided by the authors to benefit the reader, the posted materials are not copyedited and are the sole responsibility of the authors, so questions or comments should be addressed to the corresponding author.

ofz321_suppl_supplementary-appendix
