# Supplementary material for: Centralized Communication of Blood Culture Results Leveraging Antimicrobial Stewardship and Rapid Diagnostics
Source: Open Forum Infect Dis. 2019 Jul 15;6(9):ofz321. doi: 10.1093/ofid/ofz321 (PMC6736069; doi:10.1093/ofid/ofz321)
Supplement: ofz321_suppl_supplementary-appendix [file ofz321_suppl_supplementary-appendix.docx]

**Supplemental Appendix**

| **Table 1: Organisms Identified (% isolates)** | | | | | |
| --- | --- | --- | --- | --- | --- |
| **Gram negative pathogens** | | | **Gram positive pathogens** | | |
|  | Pre-intervention (2014) | Post-intervention (2016) |  | Pre-intervention (2014) | Post-intervention (2016) |
| *Escherichia coli* | 19.27 | 28.67 | MSSA | 18.8 | 13.64 |
| *Klebsiella* sp | 2.29 | 2.10 | MRSA | 11.93 | 9.09 |
| *Pseudomonas sp* | 8.26 | 8.04 | *Enterococcus faecalis* | 4.13 | 4.90 |
| *Enterobacter* sp | 3.21 | 2.45 | *Enterococcus faecium* | 0.46 | 0.35 |
| *Proteus* sp | 2.75 | 2.45 | *Streptococcus pneumoniae* | 1.83 | 4.20 |
| *Bacteroides* | 2.29 | 2.10 | *Streptococcus* species | 9.17 | 6.64 |
| Other organisms | Pre-intervention (2014): 16.24 | | Post-intervention (2016): 10.47 | | |
| MSSA= methicillin sensitive *Staphylococcus aureus*, MRSA= methicillin resistant *Staphylococcus aureus* | | | | | |

| Table 2: Median Regression Analysis for adjusted variables, 2016 vs. 2014 | | |
| --- | --- | --- |
| Outcome |  | **p-value** |
| Time to appropriate therapy, hours, median (IQR) | -0.21 (-1.28,0.85) | 0.6957 |
| Time to optimal therapy, hours, median (IQR) | -6.65 (-12.9 to -0.33) | 0.0393 |
| Time to organism ID- PCR, hours, median (IQR) | 0.0562 (-0.04 to 2.75) | 0.0562 |
| Time to organism ID- gram stain, hours, median (IQR) | 2.61 (1.09,4.14) | 0.0008 |
| Time to susceptibility, hours, median (IQR) | 7.3 (3.76,10.84) | <0.0001 |
| Inpatient cost, median, $ (IQR) | 1304.35 (-730.83-3339.52) | 0.2085 |

| Table 3: Baseline Characteristics and Outcomes- Saint Luke’s Hospital (Academic Center) | | | |
| --- | --- | --- | --- |
| Characteristic | **Pre-intervention (2014) (n=108)** | **Post-intervention (2016) (n=127)** | **p-value** |
| Age | 63.0 ± 16.9 | 63.3 ± 16.4 | 0.894 |
| Male, no. (%) | 58 (53.7) | 71 (55.9) | 0.735 |
| Patient race, no. (%)  Caucasian  Non-Caucasian | 71 (65.7)  37 (34.3) | 88 (69.3)  39 (30.7) | 0.115 |
| Comorbidities, no. (%)  Diabetes mellitus  Acute kidney injury  Chronic kidney disease  Heart failure  Chronic obstructive pulmonary disease  Myocardial infarction | 40 (37.4)  40 (37.4)  40 (37.4)  28 (26.2)  19 (17.8)  13 (12.1) | 49 (38.9)  41 (32.5)  36 (28.6)  22 (17.5)  16 (12.7)  15 (11.9) | 0.813  0.439  0.152  0.106  0.281  0.954 |
| ICU admission, no. (%) | 52 (48.1) | 52 (40.9) | 0.264 |
| Need for RRT (%) | 14 (13.1) | 13 (10.3) | 0.510 |
| Bloodstream infection source, no. (%)  Genitourinary  Line associated/foreign device/endocarditis  Intra-abdominal  Other/Unknown  Pulmonary  Skin and soft tissue/bone-joint | 25 (23.1) 33 (30.6)  9 (8.3) 15 (13.9) 11 (10.2) 15 (13.9) | 33 (26.0) 24 (18.9)  22 (17.3) 12 (9.4) 12 (9.4) 24 (18.9) | 0.110 |
| Time to appropriate therapy, hours from gram stain, median (IQR) | -12.8 (-19.5 to 2) | -13.6 (-19.8 to1.7) | 0.690 |
| Time to optimal therapy, hours from gram stain, median (IQR) | 16.2 (-6.9 to 47.5) | 6.9 (-11.9 to42.8) | 0.265 |
| ICU length of stay, days, median (IQR) | 3.5 (2.0-9.5) | 3 (2.0-7.0) | 0.264 |
| Hospital length of stay, days, median (IQR) | 8 (5.0-16.0) | 9 (6.0-14.0) | 0.536 |
| Inpatient mortality, no. (%) | 7 (6.5) | 7 (5.5) | 0.754 |
| ICU=intensive care unit, RRT=renal replacement therapy | | | |

| Table 4: Baseline Characteristics and Outcomes- Community Hospitals | | | |
| --- | --- | --- | --- |
| Characteristic | **Pre-intervention (2014) (n=110)** | **Post-intervention (2016) (n=159)** | **p-value** |
| Site  Saint Luke’s Cushing  Saint Luke’s East  Saint Luke’s North  Saint Luke’s South | 6 (5.5) 50 (45.5) 34 (30.9) 20 (18.2) | 8 (5.0) 87 (54.7) 44 (27.7) 20 (12.6) | 0.435 |
| Age | 67.7 ± 16.8 | 68.2 ± 17.0 | 0.826 |
| Male, no. (%) | 56 (50.9) | 79 (49.7) | 0.843 |
| Patient race, no. (%)  Caucasian  Non-Caucasian | 96 (87.3)  14 (12.7) | 138 (86.8)  21 (13.2) | 0.844 |
| Comorbidities, no. (%)  Diabetes mellitus  Acute kidney injury  Chronic kidney disease  Heart failure  Chronic obstructive pulmonary disease  Myocardial infarction | 41 (37.3)  25 (22.7)  24 (21.8)  19 (17.3)  18 (16.4)  13 (11.8) | 61 (38.4)  52 (32.7)  27 (23.3)  23 (14.5)  19 (11.9)  25 (15.7) | 0.855  0.075  0.779  0.532  0.301  0.365 |
| ICU admission, no. (%) | 45 (40.9%) | 49 (30.8%) | 0.087 |
| Need for RRT (%) | 4 (3.6%) | 4 (2.5%) | 0.594 |
| Bloodstream infection source, no. (%)  Genitourinary  Line associated/foreign device/endocarditis  Intra-abdominal  Other/Unknown  Pulmonary  Skin and soft tissue/bone-joint | 28 (25.5) 13 (11.8)  24 (21.8) 16 (14.5) 9 (8.2) 20 (18.2) | 60 (37.7) 13 (8.2)  19 (11.9) 16 (10.1) 14 (8.8) 37 (23.3) | 0.081 |
| Time to appropriate therapy, hours from gram stain, median (IQR) | -16.9 (-22.1 to -10.3) | -19.8 (-27.9 to -13.3) | 0.052 |
| Time to optimal therapy, hours from gram stain, median (IQR) | 9.6 (-15.2 to 48.7) | 2.5 (-22.4 to 28.1) | 0.007 |
| ICU length of stay, days, median (IQR) | 3.0 (2.0- 7.0) | 3.0 (1.0- 5.0) | 0.148 |
| Hospital length of stay, days, median (IQR) | 6.0 (4.0- 9.0) | 6.0 (4.0- 9.0) | 0.447 |
| Inpatient mortality, no. (%) | 2 (1.8) | 6 (3.8) | 0.353 |
| ICU=intensive care unit, RRT=renal replacement therapy | | | |
